# Intention-Related Natural Language Grounding via Object Affordance Detection and Intention Semantic Extraction

**DOI:** 10.3389/fnbot.2020.00026

**Published:** 2020-05-13

**Authors:** Jinpeng Mi, Hongzhuo Liang, Nikolaos Katsakis, Song Tang, Qingdu Li, Changshui Zhang, Jianwei Zhang

**Affiliations:** ^1^Institute of Machine Intelligence (IMI), University of Shanghai for Science and Technology, Shanghai, China; ^2^Technical Aspects of Multimodal Systems, Department of Informatics, University of Hamburg, Hamburg, Germany; ^3^Human-Computer Interaction, Department of Informatics, University of Hamburg, Hamburg, Germany; ^4^Department of Automation, State Key Lab of Intelligent Technologies and Systems, Tsinghua National Laboratory for Information Science and Technology (TNList), Tsinghua University, Beijing, China

**Keywords:** intention-related natural language grounding, object affordance detection, intention semantic extraction, multi-visual features, attention-based dynamic fusion

## Abstract

Similar to specific natural language instructions, intention-related natural language queries also play an essential role in our daily life communication. Inspired by the psychology term “affordance” and its applications in Human-Robot interaction, we propose an object affordance-based natural language visual grounding architecture to ground intention-related natural language queries. Formally, we first present an attention-based multi-visual features fusion network to detect object affordances from RGB images. While fusing deep visual features extracted from a pre-trained CNN model with deep texture features encoded by a deep texture encoding network, the presented object affordance detection network takes into account the interaction of the multi-visual features, and reserves the complementary nature of the different features by integrating attention weights learned from sparse representations of the multi-visual features. We train and validate the attention-based object affordance recognition network on a self-built dataset in which a large number of images originate from MSCOCO and ImageNet. Moreover, we introduce an intention semantic extraction module to extract intention semantics from intention-related natural language queries. Finally, we ground intention-related natural language queries by integrating the detected object affordances with the extracted intention semantics. We conduct extensive experiments to validate the performance of the object affordance detection network and the intention-related natural language queries grounding architecture.

## 1. Introduction

Human beings live in a multi-modal environment, where natural language and vision are the dominant channels for communication and perception. Naturally, we would like to develop intelligent agents with the ability to communicate and perceive their working scenarios as humans do. Natural language processing, computer vision, and the interplay between them are involved in the tasks for grounding natural language queries in working scenarios.

We often refer to objects in the environment when we have a pragmatic interaction with others, and we have the ability to comprehend specific and intention-related natural language queries in a wide range of practical applications. For instance, we can locate the target object “remote controller” according to the given specific natural language instruction “give me the remote controller next to the TV,” and we also can infer the intended “drinkware” from the intention-related query “I am thirsty, I want to drink some water.”

Cognitive psychologist Don Norman discussed affordance from the design perspective so that the function of objects could be easily perceived. He argued that affordance refers to the fundamental properties of an object and determines how the object could possibly be used (Norman, 1988). According to Norman's viewpoint, drinks afford *drinking*, foods afford *eating*, and readings, such as text documents are for *reading*.

When new objects come into our sight in our daily life, we can infer their function according to multiple visual properties, such as shape, size, color, texture, and material. The capacity to infer functional aspects of objects or object affordance is crucial for us to describe and categorize objects more easily. Moreover, affordance is widely used in different tasks to boost their model's performance, such as Celikkanat et al. (2015) demonstrate affordance can improve the quality of natural human-robot interaction (HRI), Yu et al. (2015) integrate affordance to improve human intentions understanding in different time period, Thermos et al. (2017) fuse visual features and affordance to improve robustness for sensorimotor object recognition, Mi et al. (2019) utilize affordance to prompt a robot to understand human spoken instructions.

Following Norman's standpoint, we generalize 10 affordances [*calling, drinking(I), drinking(II), eating(I), eating(II), playing, reading, writing, cleaning*, and *cooking*] for objects that are commonly used in indoor environments. Although drinkware and drinks can be used for drinking, drinkware affords different function to drinks, i.e., the affordance of drinkware is different from drinks. The same situation also exists between foods and eating utensils. Therefore, we utilize *drinking(I)* for denoting the affordance of drinkware, *drinking(II)* for drinks, *eating(I)* for eating utensils, and *eating(II)* for foods, respectively.

Moreover, multiple features can improve model performance to recognize objects. The texture features can be Supplementary Information for the visual representation of partially occluded objects. And according to Song et al. (2015), the local texture features can enhance the object grasping estimation performance. Motivated by the complementary nature of the multiple features, we adopt multi-visual features, the deep visual features extracted from a pretrained CNN and the deep texture features encoded by a deep texture encoding network, to learn object affordances. The primary issue of fusing multi-visual features is that the fusion scheme should preserve the complementary nature of the features. Fusing different features through naive concatenation may fail to learn the relevance of multi-features, bring about redundancies and may lead to overfitting during the training period. Consequently, in order to reserve the complementary nature of multi-visual features in the process of affordance learning, we take advantage of the interaction information between the multi-visual features, and integrate an attention network with the interaction information to fuse the multi-visual features.

Besides, inspired by the role of affordance and its applications in HRI and in order to enable robots to understand intention-related natural language instructions, we attempt to ground intention-related natural language queries via object affordance. In this work, we decompose the intention-related natural language grounding into three subtasks: (1) detect affordance of objects in working scenarios; (2) extract intention semantics from intention-related natural language queries; (3) ground target objects by integrating the detected affordances with the extracted intention semantics. In other words, we ground intention-related natural language queries via object affordance detection and intention semantic extraction.

In summary, we propose an intention-related natural language grounding architecture which is composed of an object affordance detection network, an intention semantic extraction module, and a target object grounding module. Moreover, we conduct extensive experiments to validate the performance of the introduced object affordance detection network and the intention-related natural language grounding architecture. We also implement target object grounding and grasping experiments on a robotic platform to evaluate the introduced intention-related natural language grounding architecture.

## 2. Related Work

### 2.1. Natural Language Grounding

Natural language grounding requires a comprehensive understanding of natural language expressions and images, and aims to locate the most related objects within images. Multiple approaches are proposed to address natural language grounding. Yu et al. (2016) introduce referring expression grounding which grounds referring expressions within given images via joint learning the region visual feature and the semantics embedded in referring expressions. Chen et al. (2017) present phrase grounding which aims to locate referred targets by corresponding phrases in natural language queries. These approaches need large datasets to train models to achieve natural language grounding.

Natural language grounding also attracts great interest in robotics. Thomason et al. (2017) apply opportunistic active learning to ground natural language in the home and office environment, and the presented model needs to ask human users “inquisitive” questions to locate target objects. Shridhar and Hsu (2018) employ expressions generated by a captioning model (Johnson et al., 2016), gestures, and a dialog system to ground targets. Ahn et al. (2018) utilize position maps generated by the hourglass network (Newell et al., 2016) and a question generation module to infer referred objects. Thomason et al. (2019) translate spoken language instructions into robot action commands and uses clarification conversations with human users to ground targets. However, conversation and dialog systems make HRI time-consuming and cumbersome.

Other work presents non-dialog methods to ground natural language queries. Bastianelli et al. (2016) utilize features extracted from semantic maps and spatial relationships between objects within the working environment to locate the targets for spoken language-based HRI. Alomari et al. (2017) locate target objects by learning to extract concepts of objects and building the mapping between the concepts and natural language commands. Paul et al. (2018) parse hierarchical abstract and concrete factors from natural language commands and adopts an approximate inference procedure to ground targets within working scenarios. Roesler et al. (2019) employ cross-situational learning to ground unknown synonymous objects and actions, and the introduced method utilizes different word representations to identify synonymous words and grounds targets according to the geometric characteristics of targets. These methods are proposed to ground natural language commands which embed specific target objects.

Different from the above mentioned approaches, we attempt to address intention-related natural language queries grounding without dialogs between human users and other auxiliary information. To this end, we draw support from object affordance to ground intention-related natural language instructions.

### 2.2. Object Affordance

Existing work utilizes multiple approaches to infer object affordances. Sun et al. (2014) predict object affordances through human demonstration, Kim and Sukhatme (2014) deduce affordance through extracted geometric features from point cloud segments, Zhu et al. (2014) reason affordance through querying the visual attributes, physical attributes, and categorical characteristics of objects in a pre-built knowledge base. Myers et al. (2015) perceive affordance from local shape and geometry primitives of objects. These methods adopted visual characteristics or geometric features to infer object affordances, so the scalability and flexibility of these approaches are limited.

Several recently published methods adopted deep learning-based approaches to detect object affordance. Dehban et al. (2016) propose a denoising auto-encoder to actively learn the affordances of objects and tools through observing the consequences of actions performed on objects and tools. Roy and Todorovic (2016) use a multi-scale CNN to extract mid-level visual features and combines them to segment affordances from RGB images. Unlike (Roy and Todorovic, 2016), Sawatzky et al. (2017) regard affordance perception as semantic image segmentation and adopts a deep CNN based architecture to segment affordances from weakly labeled images. Nguyen et al. (2016) extract deep features from a CNN model and apply an encoder-decoder architecture to detect affordances for object parts. Mi et al. (2019) utilize deep features extracted from different convolutional layers of pretrained CNN model to recognize object affordances, Nguyen et al. (2017) apply an object detector, CNN and dense conditional random fields to detect object affordance from RGB images.

The aforementioned work utilized geometric features or deep features extracted from a pretrained CNN to infer object affordance, and did not take into consideration that the features from another source can be applied to improve affordance recognition accuracy. Rendle (2010) propose Factorization Machines (FM), which can model interactions between different features via factorized parameters and has the capability to assess the interactions from sparse data. And (Bahdanau et al., 2015) initially present attention mechanisms to acquire different weights for different parts of input features, and can automatically search the most relevant parts to acquire better results from source features.

Inspired by Rendle (2010) and Bahdanau et al. (2015), we propose an attention-based architecture to fuse deep visual features with deep texture features through an attention network. The introduced fusion architecture takes sparse representations of the multi-visual features as input and achieves attention-based dynamic fusion for learning object affordances.

## 3. Architecture Overview

Similar to specific natural language instructions, intention-related natural language queries are also a crucial component in our daily communication. Given an intention-related natural language command, such as “I am hungry, I want to eat something,” and a working scenario which is composed of multiple household objects, the objective of intention-related natural language grounding is to locate the most related object “food” within the working scenario.

**Figure 1:**
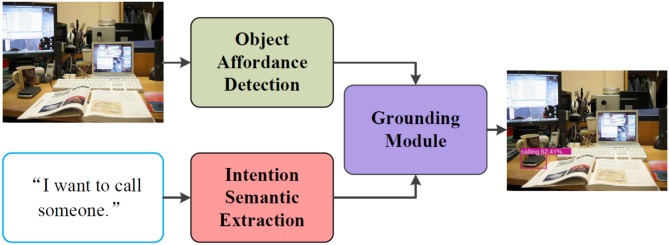
Architecture of the intention-related natural language grounding via object affordance detection and intention semantic extraction. The object affordance detection network detects object affordance from RGB images. The intention semantic extraction module calculates the different weights of each word in given natural language queries and extracts the intention semantic word. The grounding module locates target objects by combining the outputs of the object affordance detection network and the intention semantic extraction module.

In order to ground intention-related natural language queries, we propose an architecture as shown in Figure 1. In this work, we formulate the proposed intention-related natural language grounding architecture into three sub-modules: (1) an object affordance detection network detects object affordance from RGB images; (2) an intention semantic extraction module extracts semantic word from intention-related natural language instructions; (3) a target object grounding module locates intended target objects by integrating the detected object affordances with the extracted intention semantic words.

We illustrate the details of the object affordance detection in section 4, we introduce the intention semantic extraction in section 5, and we describe the target object grounding module in section 6. Moreover, we give the details of the experiments conducted to validate the performance of the object affordance detection network and the intention-related natural language grounding architecture, and outline the acquired results in section 7.

## 4. Object Affordance Detection

**Figure 2:**
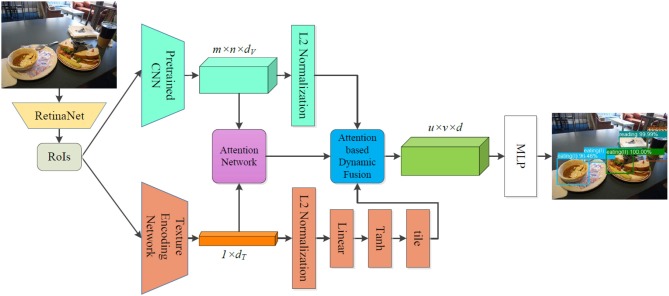
Architectural diagram of the object affordance detection via attention-based multi-visual features fusion. The RetinaNet is adopted to detect RoIs from raw images, and then for each detected RoI, the deep visual features and deep texture features are extracted by a pretrained CNN and a texture encoding network, respectively. In order to reserve the complementary nature of the different features and avoid causing redundancies during the multi-visual features fusion, an attention-based fusion mechanism is applied to fuse the multi-visual features. Through the attention-based fusion, the fused features are fed into an MLP to learn object affordances.

Following Norman's viewpoint, we generalize ten affordances for ordinary household objects, and we present an attention-based multi-visual features fusion architecture, which can be trained end-to-end, to learn the affordances. Figure 2 illustrates the details of the proposed multi-visual features fusion architecture. The presented architecture is composed of a Region of Interest (RoI) detection network (RetinaNet), a deep features extraction module, an attention network, an attention-based dynamic fusion module, and an MLP (Multi-Layer Perceptron). We adopt two different deep networks to extract multi-visual features, the attention network is employed to generate dynamic attention weights through the sparse representations of the extracted features, while the dynamic fusion module fuses the multi-visual features by integrating them with the generated attention weights, and the MLP is applied to learn the object affordances. In this section, we introduce the details of each component of the proposed architecture.

### 4.1. Deep Features Extraction

#### 4.1.1. Deep Visual Feature Extraction

RetinaNet (Lin et al., 2020) acquires better detection accuracy on MSCOCO (Lin et al., 2014) than the all state-of-the-art two-stage detectors. Considering the performance of RetinaNet, we adopt RetinaNet to generate RoIs from raw images. The deep visual feature *f*_*v*_ is extracted by a pretrained CNN for each RoI *I*_*R*_:

(1) $$\begin{array}{l} f_{v}=CNN\left(I_{R}\right) \end{array}$$

where *f*_*v*_ ∈ ℝ^*m×n×d*_*v*_^, *m* × *n* denotes the size of the extracted deep features, *d*_*v*_ is the output dimension of the CNN layer. In order to improve learning dynamics and reducing training time, we use *L*_2_ normalization to process the extracted deep visual features.

#### 4.1.2. Deep Texture Feature Extraction

Multiple presented texture recognition networks can be used to encode texture features, e.g., Cimpoi et al. (2015) generates texture features through Fisher Vector pooling of a pretrained CNN filter bank, Zhang et al. (2017) proposes a texture encoding network for material and texture recognition, the texture encoding network encodes the deep texture features through a texture encoding layer which is integrated on top of convolutional layers and is capable of transferring CNNs from object recognition to texture and material recognition. Furthermore, the texture encoding network achieves state-of-the-art performance on the material dataset MINC2500 (Bell et al., 2015). Due to the good performance of the texture encoding network introduced in Zhang et al. (2017), we select it to encode the texture feature for each detected RoI and convert the texture feature to vector **v**_***t***_:

(2) $$\begin{array}{l} {\text{v}}_{t}=TexNet\left(I_{R}\right) \end{array}$$

where **v**_***t***_ ∈ ℝ^*1×d*_*t*_^, *d*_*t*_ is the output size of the texture encoding network.

We also apply *L*_2_ normalization to process each texture vector **v**_***t***_. For modeling convenience, we utilize a single perceptron which is comprised of a linear layer and a tanh layer to transform **v**_***T***_ into a new vector:

(3) $$\begin{array}{l} {\hat{\text{v}}}_{t}=tanh\left(W{\text{v}}_{t}+b\right) \end{array}$$

where **v^**_***t***_ ∈ ℝ^1×d_*l*_^, *W* is a weight matrix and *b* is a bias vector for the linear layer, and *d*_*l*_ is the dimension of the linear layer. From Ben-Younes et al. (2017) and the experimental results, hyperbolic tangent produces slightly better results.

For fusing convenience, we adopt the tile operation to expand the texture vector $\hat{\text{v}}$_*t*_ to generate the deep texture representation *f*_*t*_ which has the same dimension with the deep visual feature *f*_*v*_, i.e., the generated *f*_*t*_ ∈ ℝ^m×n×d_*v*_^.

### 4.2. Attention-Based Multi-Visual Features Dynamic Fusion

Factorization Machines (FM) were proposed for recommendation system (Rendle, 2010), and aimed at solving the problem of feature interactions under large-scale sparse data. Given a feature vector list, FM predicts the target through modeling all interactions between each pair of features:

(4) $$\begin{array}{l} ŷ\left(x\right)=w_{0}+\overset{t}{\underset{i=1}{\sum }}w_{i}x_{i}+\overset{t}{\underset{i=1}{\sum }}\overset{t}{\underset{j=i+1}{\sum }}{ŵ}_{ij}x_{i}x_{j} \end{array}$$

where *w*_0_ ∈ ℝ is the global bias, *x*_*i*_ and *x*_*j*_ denote the *i*-th and *j*-th feature in the given feature list, *w*_*i*_ ∈ ℝ^*t*^ represents the weight of the *i*-th feature, ŵ_*ij*_ models the interaction between the *i*-th and *j*-th feature and is calculated by:

(5) $$\begin{array}{l} {ŵ}_{ij}=v_{i}^{T}v_{j} \end{array}$$

where *v*_*i*_, *v*_*j*_ ∈ ℝ^*s*^ are the sparse representations of *x*_*i*_ and *x*_*j*_, i.e., embedding vectors for the non-zero elements of *x*_*i*_ and *x*_*j*_, *s* denotes the dimension of the embedding vectors.

In light of the FM, the ŵ_*ij*_ comprises the interaction information of different features, and should be represented by the sparse non-zero elements of the different features. Formally, we extract the non-zero element set from *f*_*v*_ and **v**_***t***_, and adopt an embedding layer to acquire the sparse representations *e*_*v*_ for *f*_*v*_ and *e*_*t*_ for **v**_***t***_, respectively. We calculate the interacting matrix *k*_*vt*_ which embeds the interaction information between *f*_*v*_ and **v**_***t***_ by:

(6) $$\begin{array}{l} k_{vt}=e_{v}^{T}e_{t} \end{array}$$

where *k*_*vt*_∈ ℝ^*p*×*p*^, *e*_*v*_ and *e*_*t*_ ∈ ℝ^1×^^*p*^, *p* denotes the output size of the embedding layer.

In order to avoid causing information redundancies during features fusion, we integrate the attention mechanism with *k*_*vt*_ to complete feature fusion. By learning attention weights, the attention mechanism endows the model with the ability to emphasize the different weights of the multi-visual features during learning affordance. The attention weights can be parameterized by an attention network which is composed of an MLP and a softmax layer. The input of the attention network is the interacting matrix *k*_*vt*_, the generated weight encodes the interaction information between the different features. The attention weights τ_*att*_ can be acquired by:

(7) $$\begin{array}{l} \tau_{att}=\frac{exp\left(A_{vt}\right)}{\sum exp\left(A_{vt}\right)} \end{array}$$

and

(8) $$\begin{array}{l} A_{vt}=\alpha^{T}tanh\left(W_{att}k_{vt}+b_{att}\right) \end{array}$$

where τ_*att*_ ∈ ℝ^1×^^*p*^, *W*_*att*_, *b*_*att*_, and α are weight matrices, bias vector and model parameters for the attention network, respectively.

**Figure 3:**
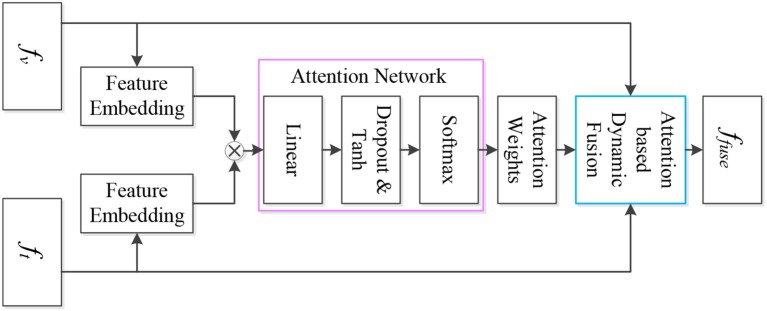
Attention-based multi-visual features fusion network. The feature embedding layers process the sparse representations of the deep visual feature and the deep texture feature, and the outputs of the feature embedding layers are applied to generate the interaction information of the multi-visual features. Subsequently, the interaction information is fed into the attention network to acquire the attention weights, which are adopted to complete attention based dynamic fusion.

By means of the learned τ_*att*_, we fuse *f*_*v*_ and *f*_*t*_ to produce the fused feature *f*_*fuse*_ to learn object affordances. The fused feature *f*_*fuse*_ is generated by:

(9) $$\begin{array}{l} f_{fuse}=\left(1-\tau_{att}\right)f_{v}⊕\left(\tau_{att}\right)f_{t} \end{array}$$

where *f*_*fuse*_ ∈ ℝ^*m×n×d*^, ⊕ denotes concatenation. Figure 3 shows the details of the attention-based multi-visual features fusion.

## 5. Intention Semantic Extraction

Each word plays a different role in representing the semantic of natural language expressions, so we argue that each word should have different weights in natural language queries to ground target objects. In order to acquire the different weights, we propose a self-attentive network to calculate the weight of each word in natural language queries. We acquire the weights in three steps. First, given a natural language sentence *S*, we tokenize *S* into words by NLTK (Perkins, 2010) toolkit, i.e., *S* = *s*_1_, *s*_2_, …, *s*_*n*_, *i* ∈ (1, *n*), n denotes the word number of *S*. Moreover, the lexical category of each tokenized word *s*_*i*_ is generated by a POS-tagger (part of speech tagger) of NLTK.

Second, we adopt GloVe (Pennington et al., 2014) to transfer *s*_*i*_ into a 300-D vector *r*_*i*_ as word representation, *r*_*i*_ ∈ ℝ^1×300^. These word representation vectors are concatenated as the representation of the sentence, i.e., *R* = (*r*_1_, *r*_2_, …, *r*_*n*_), *R* ∈ ℝ^n × 300^. We then feed the generated sentence representation *R* into the self-attentive network to calculate the weight of each word. The self-attentive network adopts an attention mechanism over the hidden vector of a BiLSTM to generate a weight score α_*i*_ for *s*_*i*_. The self-attentive network is defined as:

(10) $$\begin{array}{l} h_{t}=\text{BiLSTM}\left(R\right)\\ u_{i}=tanh\left(Wh_{t}+b\right)\\ \alpha_{i}=\frac{exp\left(u_{t}\right)}{\sum_{t}exp\left(u_{t}\right)} \end{array}$$

where *h*_*t*_ represents the hidden vector of the BiLSTM, *u*_*i*_ is the transformation vector generated by an MLP with learnable weight matrix *W* and bias vector *b*. In practice, we adopt the weight trained on the supervised data of the Stanford Natural Language Inference dataset (Conneau et al., 2017) to be the initial weight of the BiLSTM in the self-attentive network.

Finally, the sentence *S* is re-ordered according to the acquired α_*i*_, the verb with the largest weight is selected to present the semantic of intention-related instruction, and the selected verb is fed into the grounding module to complete target object grounding.

## 6. Target Object Grounding

An essential step to achieve intention-related natural language grounding is to build the mapping between the detected affordances and the extracted intention semantic words. Inspired by the Latent Semantic Analysis (LSA) which is used to measure the similarity of words and text documents meaning, we propose a semantic metric measuring based approach to build the mapping between the detected affordances and the intention-related natural language queries.

We first transfer the extracted intention semantic word and the detected affordances into 300-D vectors by GloVe, and then calculate the word semantic similarity between them to achieve target grounding. Formally, we transform the extracted intention semantic word to vector *v*_*sem*_ ∈ ℝ^1×300^, and also transfer the detected affordances into vectors *v*_*aff, i*_ ∈ ℝ^1×300^, i ∈ (1, *N*), where *N* denotes the number of detected object affordances. We calculate the semantic similarity between them by:

(11) $$\begin{array}{l} Sim\left(v_{sem},v_{aff,i}\right)=\frac{v_{sem}\cdot v_{aff,i}}{||v_{sem}|{|}_{2}\cdot ||v_{aff,i}|{|}_{2}} \end{array}$$

where ||·||_2_ denotes *L*_2_ normalization operation.

The object with the largest semantic similarity value of the intention semantic-affordance pair is selected as target. Through the semantic similarity calculation, the extracted intention semantics are mapped into the corresponding human-centered object affordance.

## 7. Experiments and Results

### 7.1. Object Affordance Detection

#### 7.1.1. Dataset

In MSCOCO (Lin et al., 2014) and ImageNet (Russakovsky et al., 2015), there are only a few indoor scenes and few objects associated with the introduced ten affordances. Therefore, we create a dataset to train and evaluate the proposed object affordance recognition architecture. The proposed dataset^1^ is composed of images collected by a Kinect V2 sensor and indoor scenes from MSCOCO and ImageNet.

**Figure 4:**
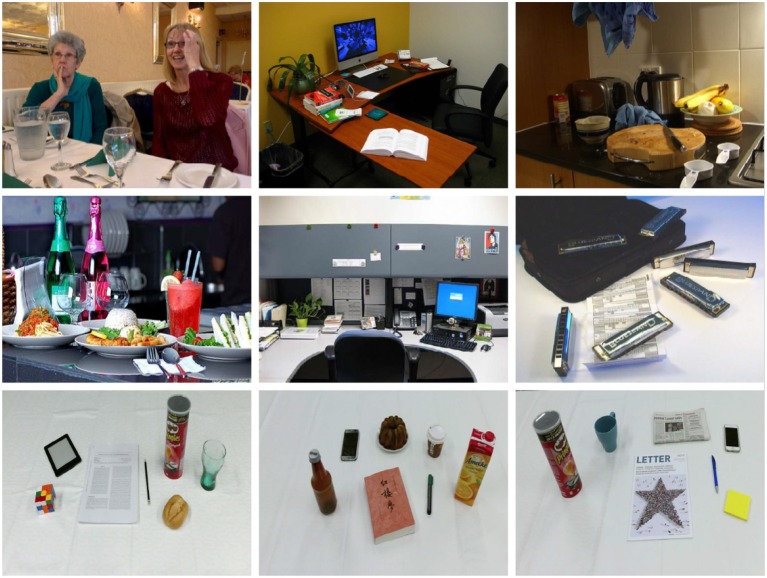
Example images from the proposed dataset. **(Top)** Images from MSCOCO. **(Middle)** Images from ImageNet. **(Bottom)** Images taken by Kinect V2.

The dataset contains in total of 12,349 RGB images and 14,695 bounding box annotations for object affordance detection (in which 3,378 annotations are from MSCOCO and ImageNet). We randomly select 56.1% regions (8,250) from the dataset for training, 22.1% regions (3,253) for validation, and the remaining 21.8% regions (3,192) for testing. Figure 4 shows some example images from the proposed dataset.

**Figure 5:**
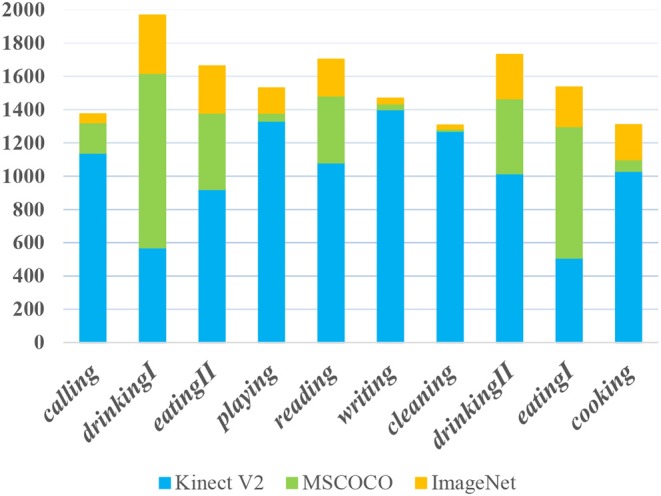
The affordance distribution in the presented dataset. Y-axis denotes the region number of each affordance.

As mentioned above, we generalize ten affordances that are related to ordinary household objects. Figure 5 illustrates the affordance distribution in the presented dataset. There are few *writing* and *cleaning* objects included in the images in the MSCOCO and ImageNet dataset, so we collect a large portion of the two categories images by a Kinect sensor.

#### 7.1.2. Experimental Setup and Results

We utilize the available source^2^ which is an implementation of RetinaNet (Lin et al., 2020) and select ResNet 50 to be the backbone to detect RoIs from RGB images. We extract the deep visual features from the last pooling layer of VGG19 (Simonyan and Zisserman, 2014) trained on Imagenet (Russakovsky et al., 2015) for each detected RoI. To produce a length-uniformed feature map for RoIs with different size, we rescaled the detected RoIs to 224 × 224 pixels. Accordingly, the dimension of the extracted deep visual feature for each RoI is 7 × 7 × 512, i.e., *f*_*v*_ ∈ ℝ^*7×7×512*^.

We adopt the deep texture encoding network (Zhang et al., 2017) trained on the material database MINC2500 to generate deep texture representations. We extract the texture features from the texture encoding layer for RoIs. The output size of the texture encoding layer is 32 × 128, so the dimension of **v**_***t***_ is 1 × 4,096. We set the output size of the single perceptron *d*_*l*_ = 512, therefore, the dimension of the transformed texture vector $\hat{\text{v}}$_*t*_ is 1 × 512. Through the tile operation, the dimension of the generated deep texture representation *f*_*t*_ ∈ ℝ^7 × 7 × 512^.

For modeling convenience, we set the size of the embedding layer to *p* = 512, the generated sparse representation for the deep visual feature and the deep texture feature, *e*_*v*_ and *e*_*t*_, are vectors with the dimension of 1 × 512, and the dimension of produced interacted matrix *k*_*vt*_ ∈ ℝ^*512×512*^. We tile the produced *k*_*vt*_ and feed it into the attention network, so the size of the generated attention weights τ_*att*_ ∈ ℝ^*1×512*^. Through the attention weights based dynamic fusion, the dimension of each produced fused feature *f*_*fuse*_ is 7 ×7 ×1,024, i.e., *f*_*fuse*_ ∈ ℝ^*7×7×1,024*^.

The fused features are fed into the MLP to learn affordances. The parameters of the MLP include: Cross Entropy loss function, Rectified Linear Unit (ReLU) activation function, and Adam optimizer. The structure of the MLP is 50176-4096-1024-10. In practice, we adopt the standard error back-propagation algorithm to train the model. We set the learning rate to 0.0001 and batch size to 32, and to prevent overfitting, we employ dropout to randomly drop 50% neurons during training.

**Figure 6:**
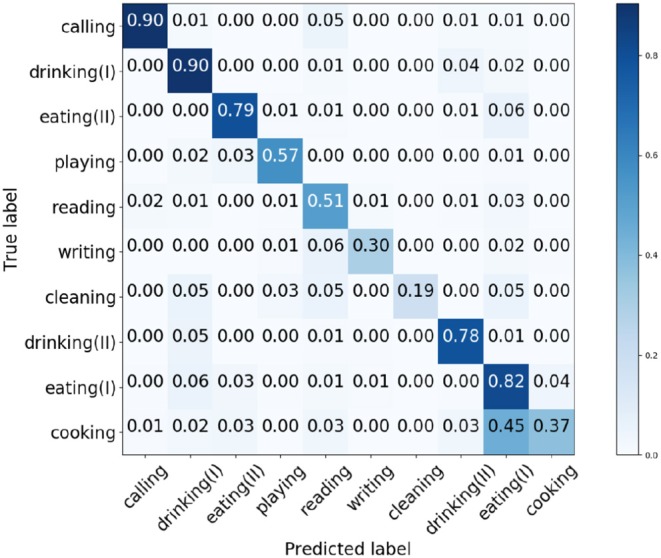
Generated confusion matrix of object affordance detection on the test set.

We train the architecture in PyTorch. After 100 epochs training, the proposed network acquires 61.38% average accuracy on the test set. Figure 6 shows the confusion matrix of the acquired results by the presented network.

**Figure 7:**
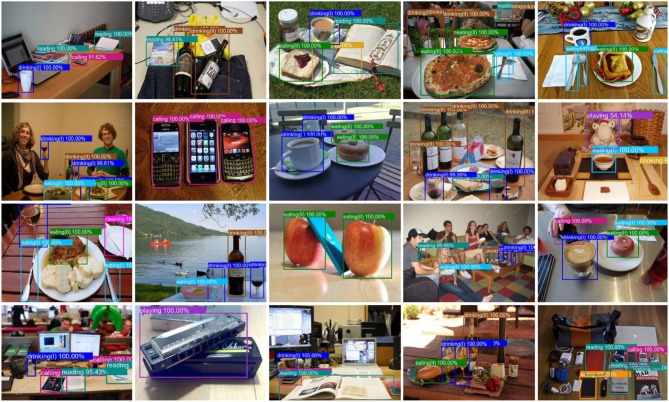
Example results of object affordance detection on the test dataset. Raw images are collected from MSCOCO and ImageNet, used with permission.

From Figure 6, the affordances *writing, cleaning*, and *cooking* have relative low accuracy compared to the other affordances. The shapes and textures of the selected objects in the three categories are significantly different from each other. Therefore, we deduce the primary cause that lead to the low accuracy of the three affordances is the great shape and texture differences, so that the similarities between the deep features in one category are difficult to generalize and learn. Figure 7 shows some acquired example results of object affordance detection on the test set.

#### 7.1.3. Ablation Study and Comparison Experiments

Except validating the attention-based multi-visual features fusion network on the presented dataset, we also adopt different features fusion approach and utilize different networks to compare the detection accuracy.

**VGG19 Deep Features**: In order to verify the effectiveness of the multi-visual features fusion for object affordances learning, we compare the results generated by the attention-base fusion network with a model trained by the deep visual features extracted from VGG 19. In this case, the deep features with shape of 7 ×7 ×512 are fed into an MLP with structure of 25088-4096-1024-10 to learn the affordances. After 100 epochs training, the generated model acquires 55.54% on the test set.

**Naive Concatenation**: For validating the performance of attention-based fusion scheme, we adopt naive concatenation to concatenate the deep visual features and the deep texture features to generate the fused representations of the multi-visual features. The concatenated features are with the shape of 7 ×7 ×1,024 and are fed into the MLP which has the same structure in the multi-visual fusion architecture to recognize affordances. After 100 epochs, the generated model acquires 58.21% on the test set.

**RetinaNet**: We directly train the RetinaNet (Lin et al., 2020) (available source^2^[2]) on the proposed dataset. For a fair comparison, the backbone also utilizes ResNet 50. After 100 epochs training, the generated model obtains 58.92% average accuracy on the test set.

**YOLO V3**: We also adopt the original pretrained weights to train YOLO V3 (Redmon and Farhadi, 2018) (available code^3^) on the dataset. After 100 epochs training, the YOLO V3 model obtain 49.63% average accuracy on the test set. Table 1 lists the results acquired by these different networks, different deep features, and different feature fusion approach.

**Table 1:** Object affordance detection results acquired by different networks, deep features and feature fusion method.

	**Attention multi-visual features fusion**	**VGG deep features**	**Naive concatenation**	**RetinaNet**	**YOLO V3**
calling	0.9036	**0.9096**	0.8723	0.7747	0.5783
drinkingI	**0.8991**	0.7785	0.8195	0.7806	0.4771
eatingII	**0.7943**	0.7658	0.7569	0.6829	0.5696
playing	0.5676	0.4791	0.5305	**0.8305**	0.7871
reading	0.5148	0.4938	0.5297	**0.6424**	0.652
writing	**0.2995**	0.2028	0.286	0.2628	0.2028
cleaning	0.1875	0.1625	0.175	**0.375**	0.3327
drinkingII	**0.7838**	0.7627	0.7248	0.6128	0.5824
eatingI	**0.8162**	0.7103	0.7049	0.6738	0.4837
cooking	0.3719	0.2893	**0.4214**	0.2562	0.2968
**Average**	**0.6138**	0.5554	0.5821	0.5892	0.4963

*The bold value of each row is the acquired best accuracy of each affordance*.

From the experimental results, it is clear that the attention-based multi-visual features fusion network acquires the higher accuracy than the VGG deep features and naive concatenation approach. Although the RetinaNet obtains 58.92% average accuracy, our attention-based fusion network acquires the best detection accuracy on five affordance categories and the best average accuracy on the test set. The results demonstrate the performance of the multi-visual features and attention-based fusion network for learning object affordances.

### 7.2. Intention-Related Natural Language Queries Grounding

In order to validate the performance of the intention-related natural language grounding architecture, we select 100 images from the introduced test dataset. To ensure the diversity of the intention-related queries, we collect 150 instructions by showing 10 participant different scenarios and ask them to give one or two queries for each image. We use the intention semantic extraction module to extract semantic words from these natural language sentences, the presented extraction module acquires 90.67% accuracy (136 correct samples in total 150 sentences).

**Figure 8:**
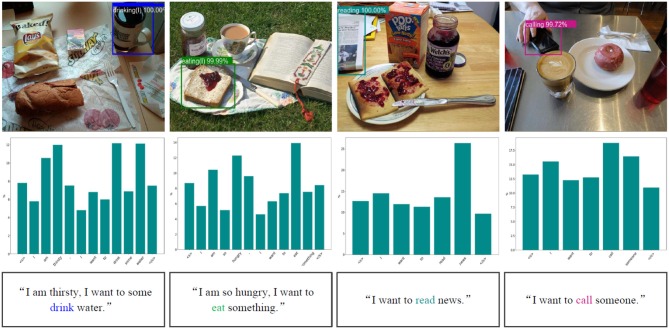
Example results of intention-related natural language query grounding. The first row lists example results of object affordance detection. The bar charts in the second row show the different weights of each word in given natural language instructions acquired by the intention semantic extraction module. <s> and </s> represent the beginning of sentence token and the end of sentence token, respectively. The third row includes the natural language queries, and the extracted intention semantic words are covered with the corresponding color of the detected affordances.

We utilize the collected images and queries to test the effectiveness of the grounding architecture. Figure 8 lists some example results of intention-related natural language queries grounding. Through analyzing the failure target groundings, we found that the performance of the grounding architecture is greatly influenced by the affordance detection.

### 7.3. Robotic Applications

We also conduct several spoken intention-related instruction grounding and target object grasping experiments on a UR5 robotic arm and a Robotiq 3-finger adaptive robot gripper platform. We first train an online speech recognizer under Kaldi (Povey et al., 2011) and translate the spoken instructions into text by the online speech recognizer, we then ground spoken intention-related queries via the introduced grounding architecture.

**Figure 9:**
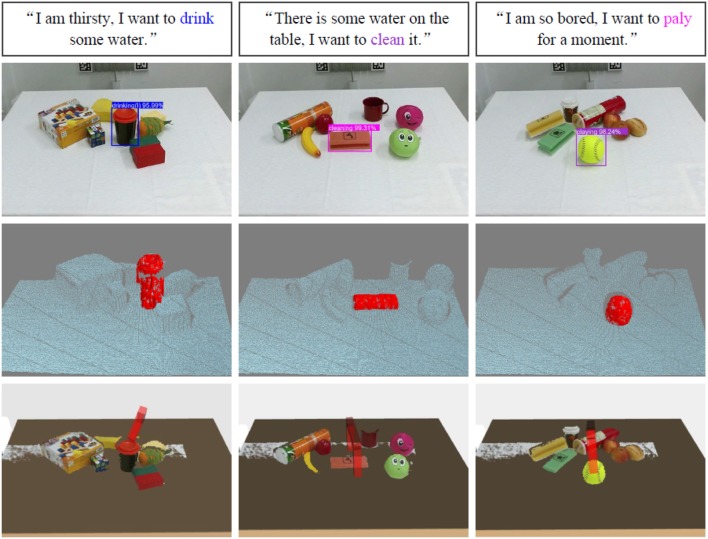
Example results of spoken natural language query groundings, point cloud segmentation, and learned target object grasping poses. The rectangles in the first row list the natural language queries, and the extracted intention semantic words are covered with corresponding color. The second row shows the results of the target object groundings. The images in the third row are point cloud segmentation by combining the bounding box values of grounded targets and the depth data acquired by a Kinect camera, and the red point clouds are the segmentations of the grounded target objects. The images in the fourth row show the grasping scenarios in MoveIt, the red grippers represent the learned best grasping poses.

In order to complete target object grasping, we combine bounding box values of the grounded target objects with depth data acquired by a Kinect V2 camera to locate the targets in 3D environments. Furthermore, we adopt the model from our previous work (Liang et al., 2019) to learn the best grasping poses. Figure 9 shows some example results of spoken instructions grounding, target objects point cloud segmentation, and learned target object grasping poses. The robotic applications video can be found in the link: https://www.youtube.com/watch?v=rchZeoAagxM.

## 8. Conclusion and Future Work

We proposed an architecture that integrates an object affordance detection network with an intention-semantic extraction module to ground intention-related natural language queries. Contrary to the existing affordance detection frameworks, the proposed affordance detection network fuses deep visual features and deep texture features to recognize object affordances from RGB images. We fused the multi-visual features via an attention-based dynamic fusion architecture, which takes into account the interaction of the multi-visual features, preserves the complementary nature of the multi-visual features extracted from different networks, and avoids producing information redundancies during feature fusion. We trained the object affordance detection network on a self-built dataset, and we conducted extensive experiments to validate the performance of the attention-base multi-visual features fusion for learning object affordances.

Moreover, we presented an intention-related natural language grounding architecture via fusing the object affordance detection with intention-semantic extraction. We evaluated the performance of the intention-related natural language grounding architecture, and the experimental results demonstrate the performance of the natural language grounding architecture. We also integrated the intention-related natural language grounding architecture with an online speech recognizer to ground spoken intention-related natural language instructions and implemented target object grasping experiments on a robotic platform.

Currently, the introduced affordance detection network learns ten affordances through fusing the deep visual features and the deep texture features. In the future, we will apply meta-learning to learn more affordances from a smaller amount of annotated images, and develop a network-based framework to learn the different contributions of the different features for object affordances learning. Additionally, we will integrate the image captioning methodology with affordance to generate affordance-aware expression for each detected region within working scenarios.

## Data Availability Statement

All datasets generated for this study are included in the article/Supplementary Material.

## Author Contributions

JM designed the study, wrote the initial draft of the manuscript, trained the object affordance detection network, completed the intention-related natural language grounding architecture, implemented and designed the validation experiments. HL completed the point cloud segmentation and grasping trajectories generation. JM and HL conducted the spoken instruction grounding experiments on the robotic platform. ST and QL provided critical revise advices for the manuscript. All authors contributed to the final paper revision.

## Conflict of Interest

The authors declare that the research was conducted in the absence of any commercial or financial relationships that could be construed as a potential conflict of interest.

## Footnotes

^1^https://tams.informatik.uni-hamburg.de/research/datasets/index.php

^2^https://github.com/fizyr/keras-retinanet

^3^https://github.com/qqwweee/keras-yolo3

## Supplementary Material

Supplementary Video 1 Robotic applications based on the proposed intention-related natural language grounding architecture.
